# Warfarin-Induced Calciphylaxis in a COVID-19 Patient

**DOI:** 10.7759/cureus.12249

**Published:** 2020-12-24

**Authors:** Fatima H Abutaki, Dunya Alfaraj, Abdullah Alshahrani, Tarek Elsharkawy

**Affiliations:** 1 Emergency, Imam Abdulrahman Bin Faisal University, Al Khobar, SAU; 2 Pathology, Imam Abdulrahman Bin Faisal University, Al Khobar, SAU

**Keywords:** calciphylaxis, warfarin, skin necrosis, covid-19, calcific uremic arteriolopathy, hypercoagulability, esrd

## Abstract

Calciphylaxis is a rare but highly fatal vascular calcification disorder with a predilection for patients with end stage renal disease (ESRD). The pathogenesis of calciphylaxis is unknown, however, several risk factors have been identified such as hypercalcemia, hyperphosphatemia, hyperparathyroidism, low serum albumin, and history of warfarin therapy. This article presents a case of calciphylaxis induced by warfarin in a COVID-19 patient.

## Introduction

Calciphylaxis also termed calcific uremic arteriolopathy (CUA) in patients with end-stage renal disease (ESRD) is a rare and life-threatening vascular calcification disorder with unclear pathogenesis. A number of studies suggest that the main pathology is hypercoagulability status which causes occlusion of small blood vessels in the subcutaneous adipose tissue and dermis. This results in painful and ischemic skin lesions [1-4].

There are few reported cases of calciphylaxis, and most of these cases were elderly patients and shared one or more of the following: female sex, obesity, impaired renal function, diabetes mellitus, hypertension, hyperparathyroidism, and use of Warfarin or calcium binders [5-8].

Patients with calciphylaxis initially present with a painful skin lesion that was described as plaque, purpura, or livedo, then rapidly progress to the stellate, malodorous ulcer with black eschars. Laboratory tests in the case of calciphylaxis are usually nonspecific, while histopathology test remains the gold standard test for definitive diagnosis. However, in cases where there is a high clinical suspicion of calciphylaxis, prompt aggressive treatment should be initiated, and histological confirmation can be reserved [1-4].

## Case presentation

A 66-year-old Saudi female presented to the ED on the 24th of October 2020 complaining of generalized abdominal pain and multiple ulcers in the left breast, lower abdomen, and right thigh. The pain started four months before the presentation; she described it as a burning sensation, and there were no aggravating or relieving factors. She had a history of COVID-19 pneumonia four months back, and after one to two weeks (the patient cannot remember exactly) these ulcers started to appear as red painful lesions, then became black with yellow to green discharge. 

She has known a case of a pulmonary embolism on warfarin for one year, which was stopped one week before the presentation at another healthcare facility. She is also a known case of uncontrolled type II diabetes mellitus on insulin for three years, ESRD on hemodialysis three times per week in the past two years. Past medical history: stroke five years back, and past surgical history: hemithyroidectomy 10 years ago and since then she was kept on thyroxin.

**Figure 1:**
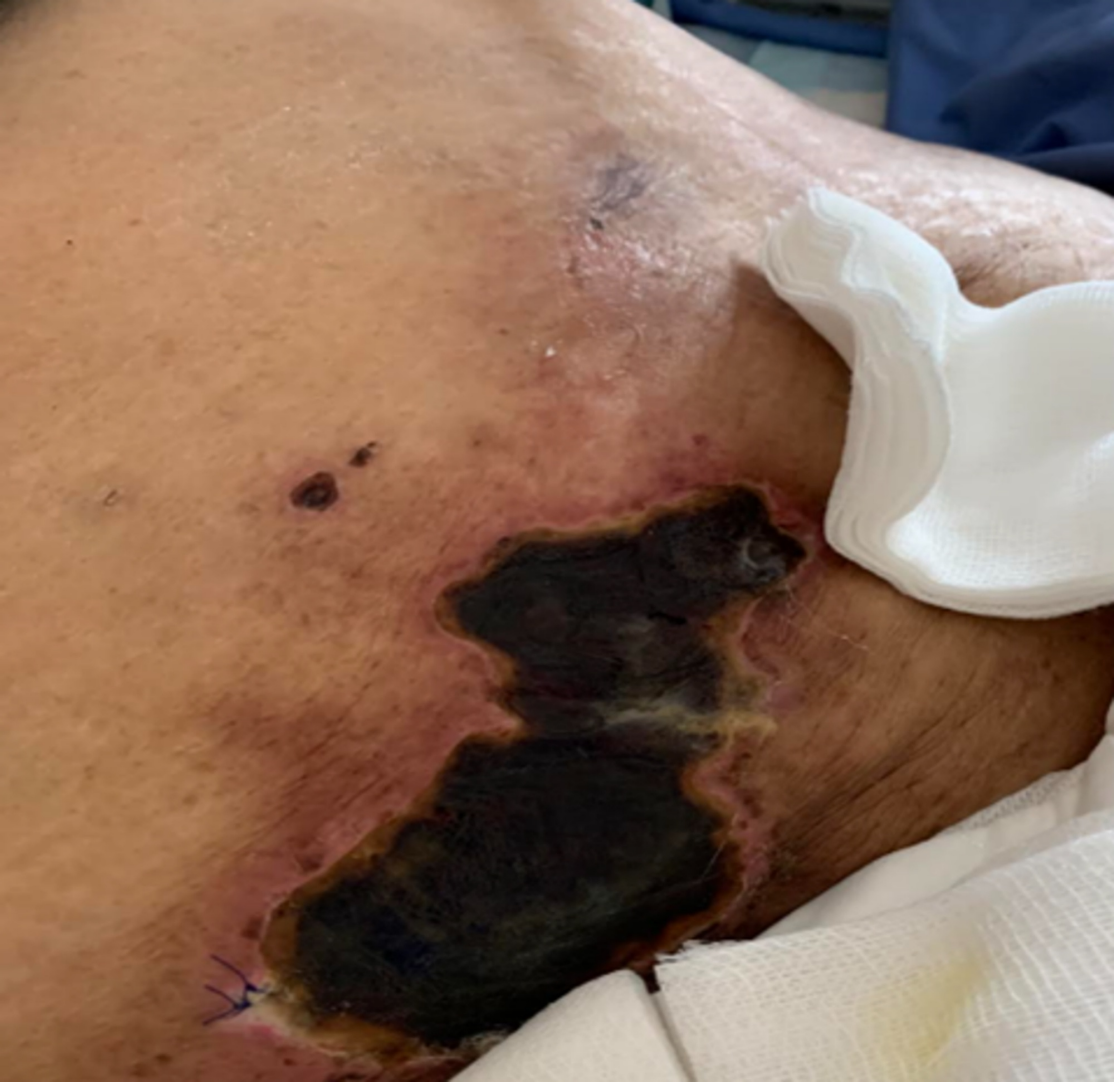
Skin ulcer in the right lower abdomen with pus discharge.

Upon general examination, the patient was hemodynamically stable, conscious, alert, and oriented, and the systemic examination was unremarkable. There were multiple skin ulcerations in the left breast, right lower abdomen (as shown in Figure 1), and the right thigh extending up to the groin. These ulcers were red to black with pus discharge.

There were two main differential diagnoses, first one was warfarin-induced skin necrosis; this was excluded because this condition should be improved after discontinuation of warfarin. Another differential diagnosis was COVID-19-related skin necrosis which was differentiated from calciphylaxis after histopathology results [1, 9]. Other differentials were excluded such as atherosclerotic vascular disease, venous stasis ulcer, cholesterol embolization, necrotizing vasculitis, and livedoid vasculopathy [10-11].

**Figure 2:**
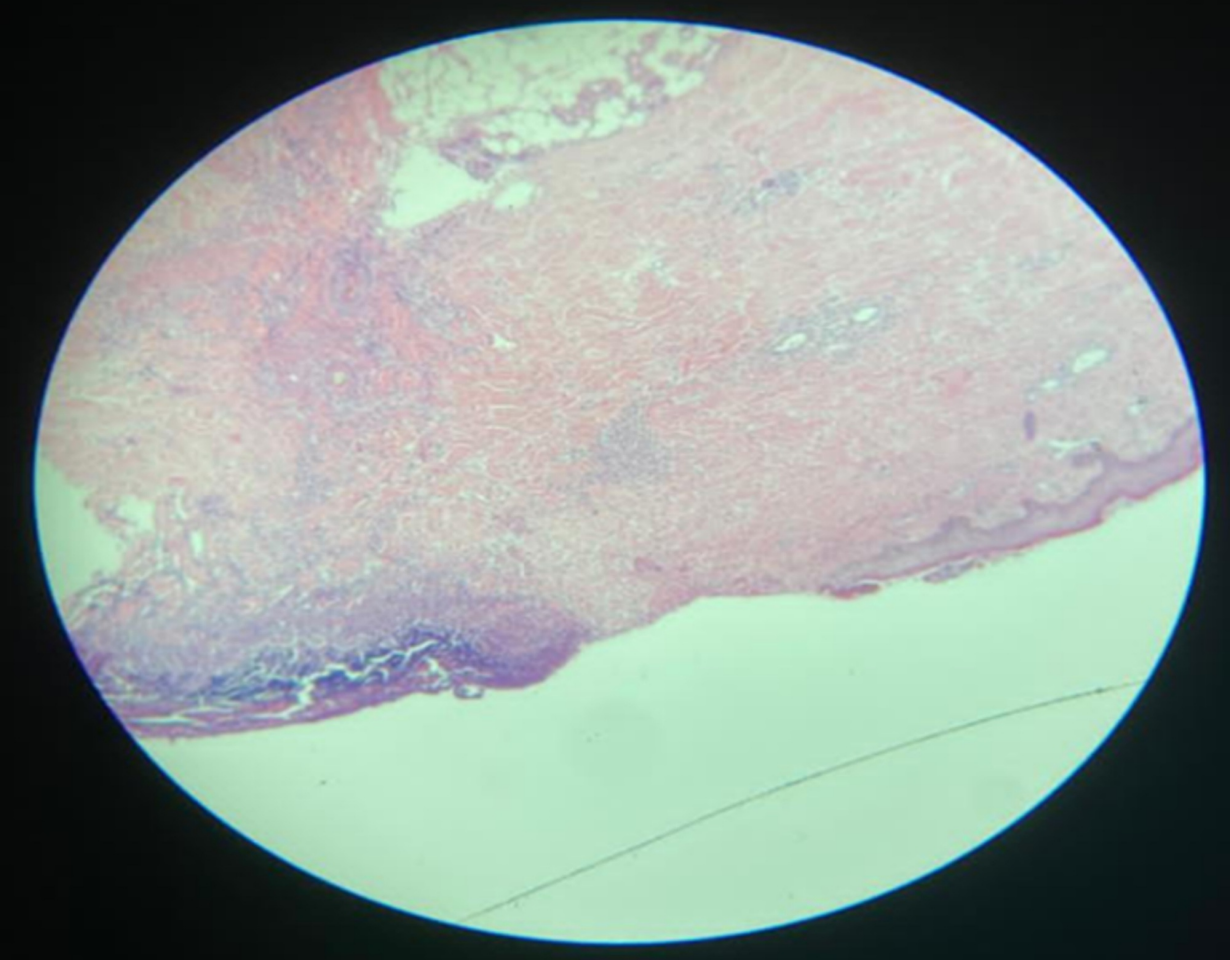
Hematoxylin and eosin stain (40X): epidermal necrosis with diffuse superficial and deep dermatitis; an inflammatory process at the base with prevalence of neutrophils up to pustular formation.

**Figure 3:**
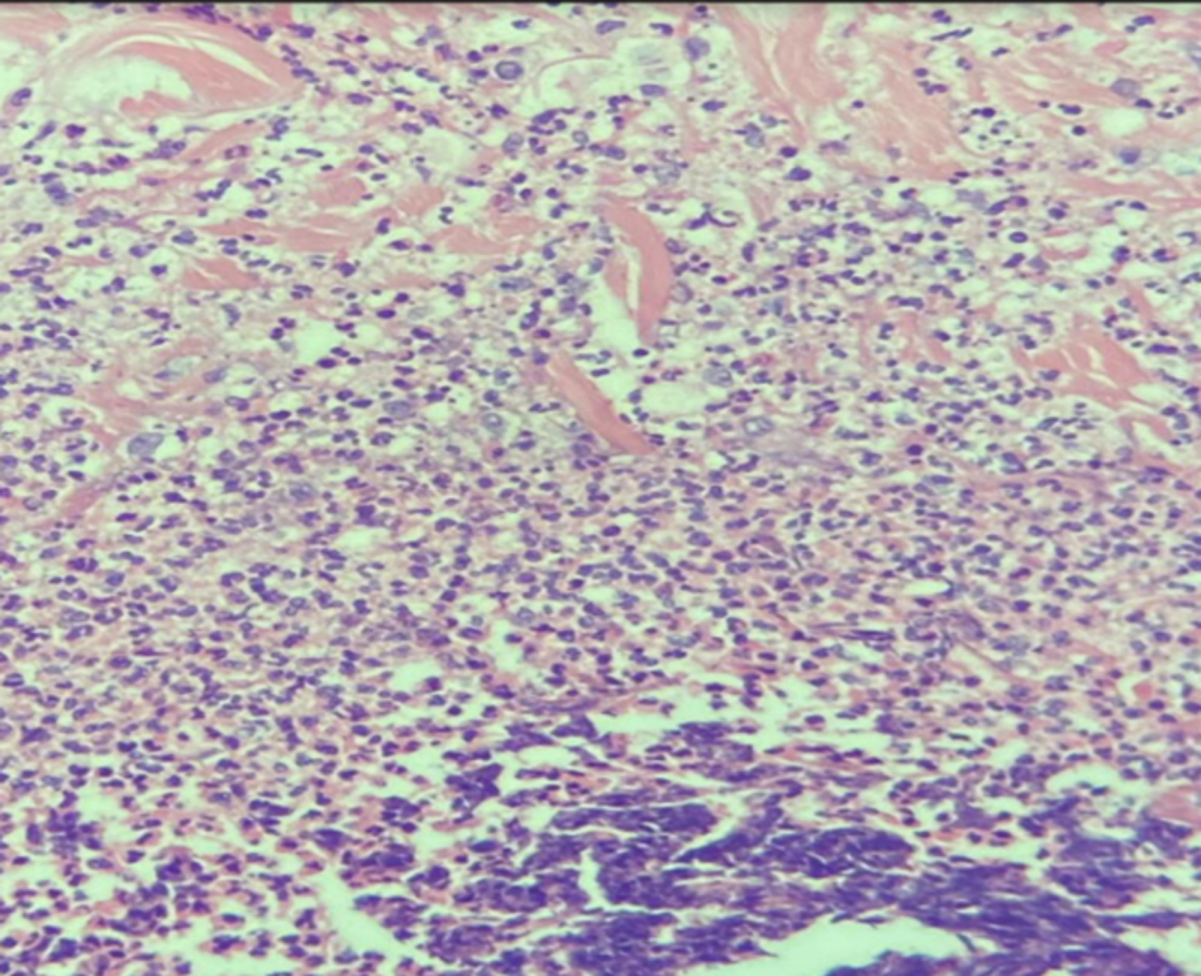
Hematoxylin and eosin stain (100x): epidermal necrosis with diffuse superficial and deep dermatitis; an inflammatory process at the base with the prevalence of neutrophils up to the pustular formation.

**Figure 4:**
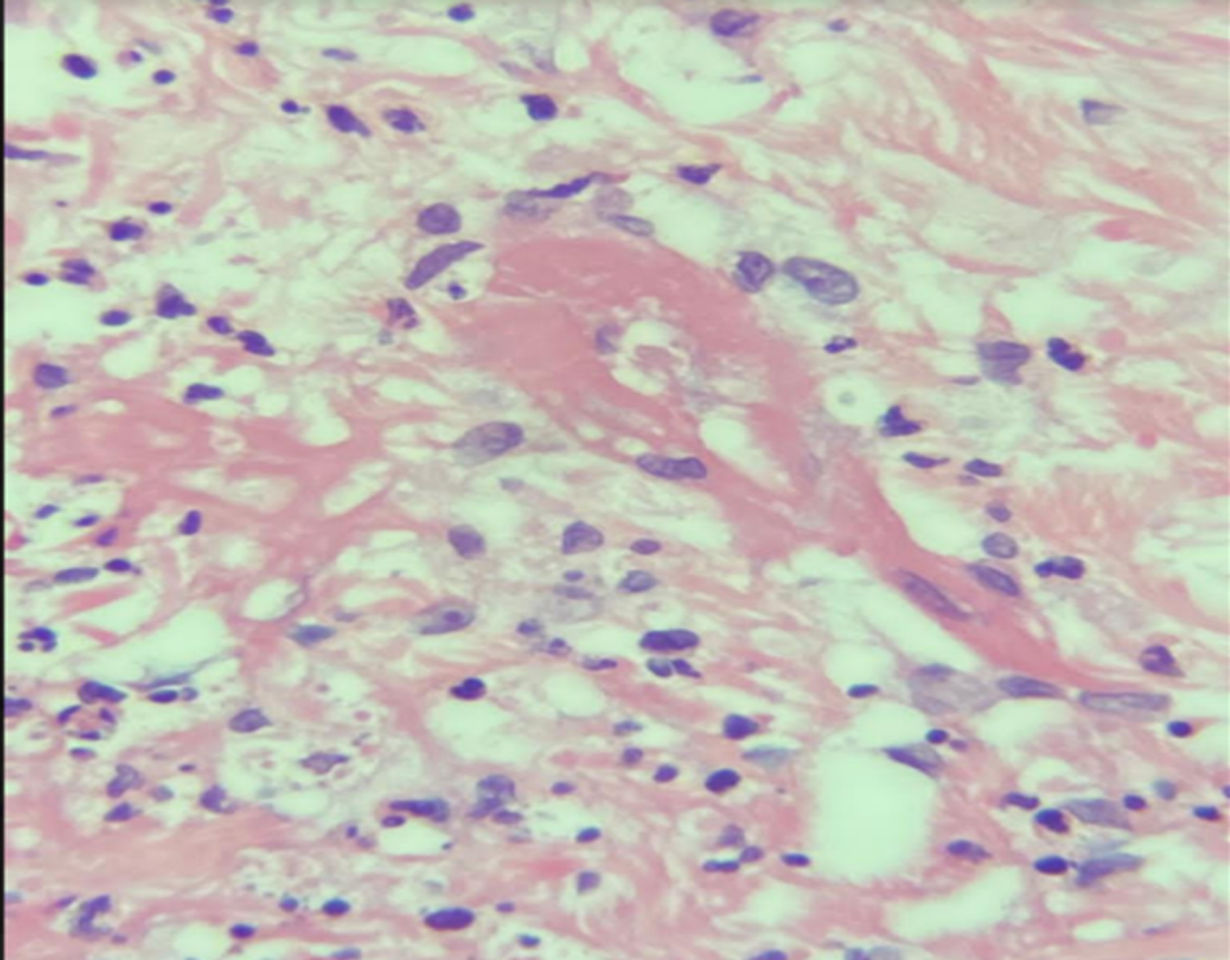
Hematoxylin and eosin stain (200x): fibrinoid necrosis of dermal blood vessel with leukocytoclastic vasculitis.

**Figure 5:**
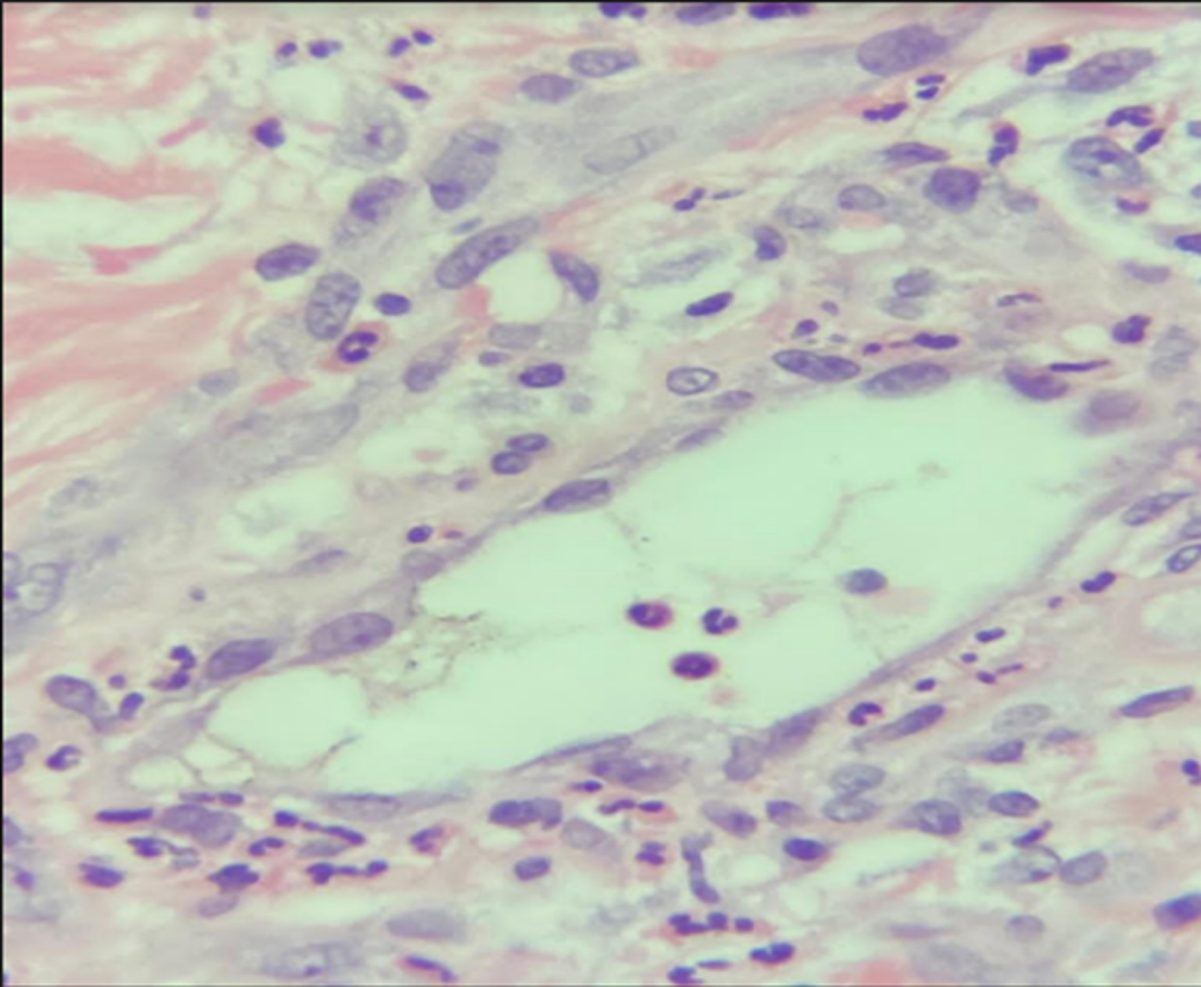
Hematoxylin and eosin stain (200x): fibrinoid necrosis of dermal blood vessel with leukocytoclastic vasculitis.

Initial laboratory tests were done to investigate other differential diagnoses (Table 1). Microbiology tests were conducted and showed: *Pseudomonas aeruginosa* from the wound culture, vibrio cholera, and multiple drug resistance (MDR) *P. aeruginosa* from tissue culture, but there was no growth from stool culture. For initial imaging, enhanced CT was done and revealed an osteoporotic fracture of L1, and skin thickening, and subcutaneous fat at the right lower abdomen with no intra-abdominal collection. Moreover, a pulmonary angiography scan was done and excluded pulmonary embolism. Tissue biopsy confirmed the diagnosis of calciphylaxis as it showed markedly necrotic connective and fibrofatty tissue with dystrophic calcification and autolytic changes without signs of malignancy. Figures 2-3 demonstrate epidermal necrosis with diffuse superficial and deep dermatitis and inflammatory process at the base with a prevalence of neutrophils up to the pustular formation. Figures 4-5 show fibrinoid necrosis of dermal blood vessels with leukocytoclastic vasculitis.

The patient was admitted to the general ward and managed primarily by supportive measures while waiting for the tissue biopsy results. These measures are as follows: regular hemodialysis, diabetic diet, daily wound care, pain management, empirical antibiotic, and adjustment of home medications. Following the culture sensitivity, the patient was shifted to a culture-sensitive antibiotic. After that, she was clinically stable, and her pain subsides, but the skin lesions persist. Therefore, she was discharged on the 1st of December 2020 with outpatient clinic follow-up.

 

**Table 1:** Patient's laboratory investigations. CBC, complete blood count; LFT, liver function test; SGOT, serum glutamic-oxaloacetic transaminase; SGPT, serum glutamic-pyruvic transaminase; LDH, lactate dehydrogenase; GGTP, gamma-glutamyl transferase; RFT, renal function test; BUN, blood urea nitrogen; CRP, C-reactive protein; ESR, erythrocyte sedimentation rate; PT, prothrombin time; INR, international normalized ratio; aPTT, activated partial thromboplastin time

Investigation	Reference range	Patient result
Complete blood count		
White blood cells count	4–11 k/uL	14.6 k/uL
Hemoglobin	12–16 g/dL	12.3 g/dL
Platelets	140–450 k/uL	344 k/uL
Liver function test		
Indirect bilirubin	0.2–1.2 mg/dL	0.3 mg/dL
Direct bilirubin	0.05–0.2 mg/dL	0.09 mg/dL
Total bilirubin	6.4–8.2 g/dL	7.3 g/dL
Albumin	3.4–5.0 g/dL	2.7 g/dL
Alkaline phosphatase	46–116 U/L	156 U/L
SGOT	15–37 U/L	9 U/L
SGPT	14–63 U/L	15 U/L
LDH	81–234 U/L	156 U/L
GGTP	5–55 U/L	64 U/L
Renal function test		
BUN	7–18 mg/dL	28 mg/dL
Creatinine	0.6–1.0 mg/dL	4.73 mg/dL
Na	136–145 mEq/L	134 mEq/L
K	3.5–5.1 mEq/L	4.2 mEq/L
Cl	21–32 mEq/L	95 mEq/L
CO2	35–45 mEq/L	26 mEq/L
Anion gap	8–12	13.00
Inflammatory markers		
C-reactive protein	0.05–0.3 mg/dL	19.2 mg/dL
Erythrocyte sedimentation rate	0–20 mm/h	100 mm/h
Procalcitonin	<= 0.1 ng/mL	6.39 ng/mL
Coagulation profile		
Prothrombin time	12.9–15.9 s	22.4 s
INR	1–2	1.95
Prolonged partial thromboplastin time	25.6–42.3 seconds	50.7 s
Venous blood gas		
pH	7.35–7.45	7.387
PCO2	35–45 mmHg	49.8 mmHg
PO2	83–108 mmHg	20.4 mmHg
HCO3	22–26 mmol/L	26.4 mmol/L
Electrolytes		
Ca	8.5–10.1 mg/dL	9.7 mg/dL
Mg	1.8–2.4 mg/dL	1.8 mg/dL
PO4	2.6–4.7 mg/dL	4.6 mg/dL
Blood glucose		
Random blood glucose	70–140 mg/dL	254 mg/dL
Glycated hemoglobin (HA1c)	4%–6%	9.6%

## Discussion

Based on the clinical data, and the histopathology findings this patient was diagnosed with warfarin-induced calciphylaxis, which was precipitated by COVID-19. This suspicion was raised at the beginning, because of the patient’s risk factors, which were as follows: female sex, obesity (BMI 45.8), thrombophilia, ESRD on hemodialysis, type II diabetes mellitus, and use of warfarin [12-13].

Other risk factors that are frequently implicated in patients with calciphylaxis include rapid weight loss, hepatobiliary disease, hypoalbuminemia, vitamin K deficiency, hypercalcemia, hyperphosphatemia, and hyperparathyroidism or medical treatment such as calcium-based phosphorus binder and vitamin D [1-3, 12-13]. 

Clinical presentation of calciphylaxis can be classified into nonulcerated lesions (early-stage) and ulcerated lesions (late-stage). The affected areas can be peripheral adipose tissues (e.g., digits and toes) or central (e.g., abdomen and thigh) which are more common in patients with high body mass index (BMI) and ESRD. This patient had a late-stage presentation with central lesions mainly, this indicated poor prognosis [1-2].

Skin biopsy is considered the gold standard diagnostic test for calciphylaxis, which demonstrates calcified blood vessels with or without fibrosis. CT scans and laboratory tests are also helpful for an initial assessment to exclude other differential diagnoses as mentioned earlier [1, 14].

Calciphylaxis is treated supportively by the elimination of risk factors, relieving of the pain, adequate protein intake, and wound care. Intravenous sodium thiosulfate is one of the most common therapeutic interventions for calciphylaxis. It is an antioxidant and vasodilator medication that is used to inhibit calcification of the vascular wall. Some medications have been described in case reports that may have potential benefit in treating calciphylaxis such as vitamin K, a low dose of tissue plasminogen activator infusion, bisphosphonates, low-density lipoprotein apheresis, and kidney transplantation [4, 15-17].

This case is reported because calciphylaxis remains a relevant subject for medical research as the pathogenesis is not yet clear, and the mortality rate reaches up to 80%. Besides, this case concurrence with COVID-19 raised questions for further research about skin manifestation in COVID-19 infection. 

## Conclusions

Understanding the risk factors of calciphylaxis is necessary both in the diagnosis and management of this rare and life-threatening condition. Given its high mortality and poor prognosis, early diagnosis and comprehensive management by a multidisciplinary team are important.
